# Assessing patent ductus arteriosus in preterm infants from standard neonatal intensive care monitoring

**DOI:** 10.1007/s00431-021-04311-9

**Published:** 2021-11-08

**Authors:** Charalampos Kotidis, David Wertheim, Michael Weindling, Heike Rabe, Mark A. Turner

**Affiliations:** 1 Department of Women’s and Children’s Health, University of Liverpool, Liverpool Health Partners, Liverpool, UK; 2 University of Liverpool, Liverpool Womens Hospital, Crown Street, L8 7SS Liverpool, UK; 3 Faculty of Science, Engineering and Computing, Kingston University, Surrey, UK; 4 Academic Department of Paediatrics, Brighton and Sussex Medical School, University of Sussex, Brighton, UK

**Keywords:** Echocardiography, Biomarkers, Haemodynamics, Patent ductus arteriosus, Preterm infants

## Abstract

Monitoring patent ductus arteriosus (PDA) in premature infants is currently performed intermittently using echocardiography which requires considerable expertise. The aim of this pilot study was to investigate whether PDA status could be assessed from standard neonatal intensive care monitoring. Electrocardiography (ECG) and blood pressure (BP) waveforms were acquired from extremely preterm infants using standard neonatal monitors. We developed software using MATLAB to analyse ECG and BP waveforms and their interrelationships in terms of pulse transit time (PTT) and pulse wave velocity (PWV). The times from peak systolic BP to diastolic trough (BPFt) and from the diastolic trough to peak systolic BP (BPRt) were also calculated. PTT, BPFt and BPRt were normalised for heart rate (HR) termed NPTT, NBPFt and NBPRt, respectively. ECG, invasive aortic BP monitoring and echocardiography were performed in 14 preterm infants < 29 weeks’ gestation in the first 3 days after birth. The median (range) birth weight of the infants was 0.90 (0.48–1.31) kg, gestation 26.6 (24.0–28.7) weeks, PDA diameter 1.6 (0.8–3.6) mm and mean BP 32 (16–40) mmHg. We found a significant positive correlation between PDA diameter and NPTT (*r* = 0.69, *P* = 0.007) as well as NBPFt (*r* = 0.65, *P* = 0.012) and NBPRt (*r* = 0.71, *P* = 0.005). No relationship was found between PDA diameter and pulse pressure.

*Conclusions*: Interrelationships between ECG and BP traces as well as BP waveform time analysis are straightforward to measure and associated with PDA diameter. The results of this pilot study suggest that this approach may help provide biomarkers for continuous monitoring PDA diameter and function.

**Table:** 

**What is Known:** *• Patent ductus arteriosus (PDA) in premature infants is associated with increased risk of developing chronic lung disease, necrotising enterocolitis and cerebral injury.* *• Currently PDA is assessed intermittently using echocardiography which requires considerable expertise and sometimes is not well tolerated by critically ill preterm infants.*	
**What is New:** *• Blood pressure (BP) and ECG waveform interrelation and BP trace time analysis, taking account of heart rate, relate to PDA diameter.* *• ECG and BP waveform phase difference as well as BP waveform time analysis may be useful in the continuous assessment of PDA function. *

**Supplementary information:**

The online version contains supplementary material available at 10.1007/s00431-021-04311-9.

## Introduction

Patent ductus arteriosus (PDA) has significant effects on cardiac and aortic haemodynamics as blood flow through the PDA reduces flow to the systemic circulation and overperfuses the lungs. PDA is associated with many neonatal comorbidities [1, 2]. Effective treatments for PDA are available [3]; however, there is no consensus for the definition of the haemodynamically significant PDA, and the decision for treatment is based on a combination of echocardiographic and clinical criteria. Echocardiography, the gold standard for PDA assessment, is assessed intermittently, requires considerable expertise and is sometimes not well tolerated by extremely preterm infants (EPIs) [4].

Pulse transit time (PTT) is of value in monitoring blood pressure (BP) changes in adults [5]. Different methods are used to measure PTT, but typically the time difference between ECG and pulse oximetry plethysmogram traces are determined with an oximeter probe placed on limbs [6]; the difference in time between the ECG R wave and the midway of the following corresponding plethysmographic trace upswing is measured [7]. Pulse wave velocity (PWV) can be calculated from the physical length between the heart and the oximeter probe divided by PTT. Aortic PWV is a marker for cardiovascular events in adults [8] as well as having use in monitoring BP [9].

In most UK neonatal intensive care units, EPIs may have an umbilical artery catheter (UAC) for BP monitoring in the days after birth, the tip of which lies in the descending aorta close to the ductus arteriosus insertion. However, there has been little reported use of PWV or PTT in preterm infants probably in part due to the high heart rate (HR) and small physical lengths making the measurement precision have greater influence. As high-resolution digital recording of ECG and BP is now available, PWV and PTT in neonates can be more easily determined. This study aimed to investigate whether there are changes in PTT, PWV and BP wave characteristics in EPIs associated with PDA diameter.

## Methods

This was a nested cohort study within a prospective observational study investigating whether there is a relation between PDA and brain haemodynamics (North West Lancaster ethics committee, REC reference: 14/NW/1274). All neonates admitted to Liverpool Women’s Hospital between 24^+0^- and 28^+6^–week gestation and postnatal age ≤ 72 h were recruited during the period between August 2015 and December 2016 with parental consent given either pre- or postnatally. Only babies with recorded BP waveform from a UAC were entered into the current study.

The exclusion criteria were non-viability, chromosomal anomalies or other malformations likely to affect cardiovascular adaptation and intraventricular haemorrhage grades 3–4 in the cranial ultrasonography after birth.

### Clinical physiological monitoring

ECG and BP were monitored for clinical reasons in accordance with standard neonatal intensive care using Philips IntelliVue MX800 patient monitors (Philips Healthcare, UK). The data were recorded from the monitors by interfacing with a laptop via Bluetooth using IxTrend software (ixellence GmbH, Wildau, Germany) and shortly before echocardiography on that day.

### Invasive BP monitoring

Invasive BP data were only captured when BP monitoring was clinically indicated. A 3.5F UAC (Vygon, Swindon, UK) was positioned between the sixth and tenth thoracic vertebra and connected to an electronic pressure transducer via a 38-cm-long rigid plastic extension catheter tubing. The distance from the BP transducer was thus made up of 37 cm (UAC length) + 38 cm (extension length) giving a total of 75 cm distance. The ECG and BP waveforms on the monitor screen were visually assessed to ensure good quality signal with no apparent movement artefact. Indicators of poor BP signal quality were an abnormal shape waveform (indicating damping with air in the circuit) or a low pulse pressure (indicating partially blocked UAC); the BP transducer was positioned at the level of the heart. A low pass filter cut-off frequency at 12 Hz was applied by the monitor to the BP waveform and the output exported with 125 Hz sampling frequency.

### Electrocardiogram (ECG)

Standard neonatal ECG for clinical monitoring was applied and lead I selected. ECG and BP monitoring data were downloaded from monitors and stored as spreadsheet files using the IxTrend software. The ECG waveform data were acquired with 500 Hz sampling rate (filter range 0. to 55 Hz).

### Assessment of ECG and BP interrelation and BP waveform measurements

**Fig. 1:**
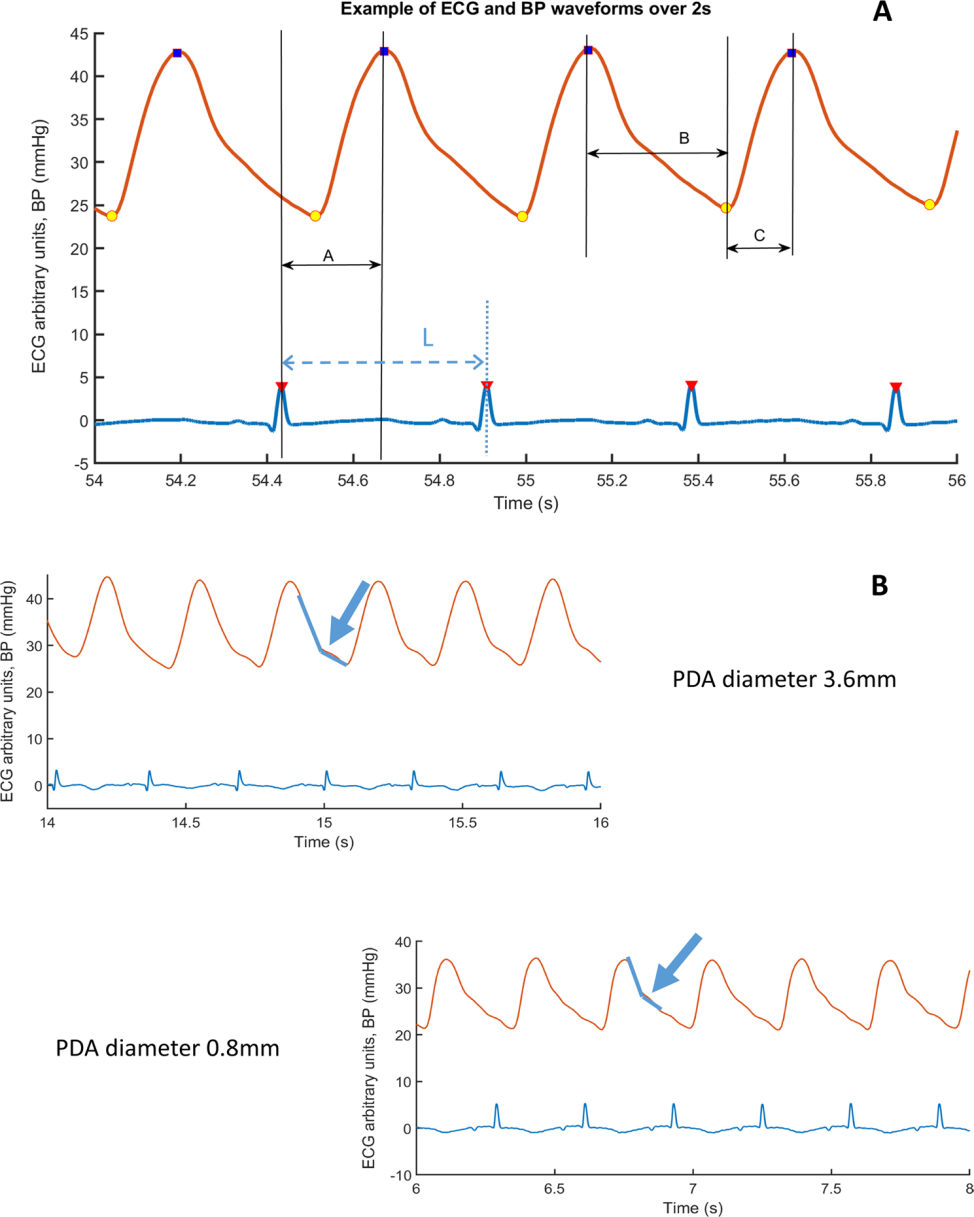
**A** Cardiac cycle length (L) is readily measured from the ECG as well as the time difference PTT (A) between the ECG R wave (red triangle) and the following BP systolic peak (blue square). The ratio PTT:L gives a dimensionless index NPTT that is normalised to HR. The difference in time between peak systole and end diastole (BPFt) is shown as B. The difference in time between end diastole and the following peak in systole (BPRt) is shown as **C**. The surrogate for isovolumic contraction time (MIVCT) was calculated as PTT (A)–BP rise time (C) in a single cardiac cycle. **B** Two examples of ECG (lower) and BP (upper) waveforms of two seconds duration with different PDA diameters. Infants with larger PDA had the dicrotic notch displayed towards the end of the dicrotic limb which can be attributed to the lower overall arterial resistance due to higher run off from the large PDA

Using MATLAB (The MathWorks Inc., USA), we developed software to read, display and analyse the ECG and BP spreadsheet files collected using IxTrend. PTT was defined in our study as the difference in time between the ECG R wave and the following systolic BP peak (Fig. 1). The systolic BP peak was used to enable precise identification of a consistent point in the cardiac cycle; this is important because of the high HR in preterm neonates.

For PWV the distance along the blood vessels from the aortic valve to the UAC tip was estimated. As the catheter tube connecting to the pressure transducer is rigid, it was assumed this would have negligible effect on PWV calculation; the catheter position was confirmed by X-ray imaging. The aortic valve is radio translucent with no clear landmark to identify it; from adult data, the relation of the aortic valve to vertebral level varies around the middle third of the seventh thoracic vertebra [10]. There are no corresponding data for preterm infants, and hence, we reviewed CT scans from the term infants with cardiac conditions and found that the relation of the aortic valve to the vertebral level varied but mostly was around the 6th thoracic vertebra. The distance of the catheter tip from the assumed position of the aortic valve could thus be traced using PACS software (Phillips, London, UK).

As PTT and PWV are affected by HR, PTT was expressed as a fraction of the RR interval to normalise for HR. Thus, NPTT is the difference in time between the ECG R wave and the following systolic BP peak expressed as the proportion of one cardiac cycle, R–R interval length (L) (Fig. 1A); NPTT is thus the ratio PTT/L.

The median HR, PTT and NPTT over 10 s were computed by our custom software in two sections with little or no artefact. The sections were 10 min apart unless there was artefact in which case the second section was as close to 10 min later as possible. The software also calculated the minimum and maximum BP for each cardiac cycle as well as the pulse pressure and mean BP calculated based on the systolic and diastolic values (diastolic + 1/3 pulse pressure) [11]. The software also calculated the time from peak systolic BP to the trough in diastole termed BP fall time (BPFt) and the time from the BP trough in diastole to the following peak systolic BP, termed BP rise time (BPRt). As the measurements could potentially be affected by HR, the data were also normalised with respect to HR, i.e. the proportion of one cardiac cycle, giving three new measurements termed NPTT, NBPFt and NBPRt, respectively. To assess which component of cardiac cycle is mainly affected by PDA, a surrogate of isovolumic contraction time was calculated by deducting BPRt from PTT termed MIVCT and expressed as a ratio normalised to HR (NMIVCT).

### Echocardiography and cerebral Doppler blood flow velocity

Echocardiography and cerebral ultrasonography were performed daily in the first 3 days after birth using a Vivid-E9 machine with a 12 MHz phased array probe (GE Medical, Milwaukee, USA). PDA diameter was measured from the high parasternal view at the narrowest point [12]. Multiple echocardiographic parameters were measured as previously described [13]. Cerebral Pourcelot resistance index (PI) from Doppler ultrasound measurements was computed as it is based on the blood flow velocity waveform [14]. Images and videos were acquired and analysed offline by a single observer (CK).

### Statistics

Statistical analysis was performed using Minitab v19 (Minitab LLC., USA). Data were tested for consistency with a normal distribution using the Ryan-Joiner test and parametric or non-parametric statistics used as necessary. Median and range were used to describe summary demographics. Bland and Altman plots [15] were used to assess the repeatability of the data separated by 10 min. Pearson’s correlation coefficient was used to assess the relationship between the aortic biomarkers with demographic and cardiovascular parameters. In view of multiple comparisons, the Benjamini and Hochberg procedure [16] was applied with 10% false discovery rate.

## Results

Fourteen infants were studied in the first 3 days after birth, and a single measurement was analysed for each infant. The demographic details of our population and a summary of measurements are shown in Table 1. In summary the median (range) birth weight (BW) was 0.90 (0.48–1.31) kg, gestation 26.6 (24.0–28.7) weeks, PDA diameter 1.6 (0.8–3.6) mm, HR 147 (111–191)/min and mean BP at the measurement time was 32 (16–40) mmHg. Four neonates received inotropes (dopamine and/or dobutamine); of these one subsequently had a severe intraventricular haemorrhage and three later died. In one recording, the infant was clearly hypotensive with systolic BP 25 mmHg and diastolic 11 mmHg, mean 16 mmHg. There was a significant relationship between PTT and gestation (*r* = 0.55, 95% CI (0.03, 0.84), *P* = 0.040), but not with BW (*r* = 0.39, 95% CI (−0.17, 0.76), *P* = 0.165). Furthermore, there was a significant relationship between NPTT and BW (*r* = 0.57, 95% CI (0.05, 0.84), *P* = 0.035), but not gestation (*r* = 0.40, 95% CI (−0.16, 0.77), *P* = 0.154).

**Table 1:** Summary of demographic data and range of measurements. *PTT* pulse transit time, *PWV* pulse wave velocity, *PDA* patent ductus arteriosus, *BP* blood pressure, *UAC* umbilical artery catheter

Patients *n* = 14	Median	Range (min to max)
Birth weight (kg)	0.9	0.48–1.31
Gestation (weeks)	26.6	24.0–28.7
PTT (s)	0.2	0.14–0.26
PWV (m/s)	2.08	1.30–3.38
Normalised PTT (NPTT)	0.487	0.444–0.586
PDA (mm)	1.6	0.8–3.6
UAC distance from assumed AoV (cm)	43	26–53
Heart rate (beats/min)	147	110–191
Mean BP (mmHg)	32	16–40
Cerebral Pourcelot resistance index (PI)	0.72	0.58–1.00

Visual analysis of BP waveform morphology indicated changes associated with PDA diameter; infants with large PDA diameter had the dicrotic notch towards the end of the dicrotic limb and had a smoother dicrotic limb without superimposed pressure perturbations (Fig. 1B).

**Fig. 2:**
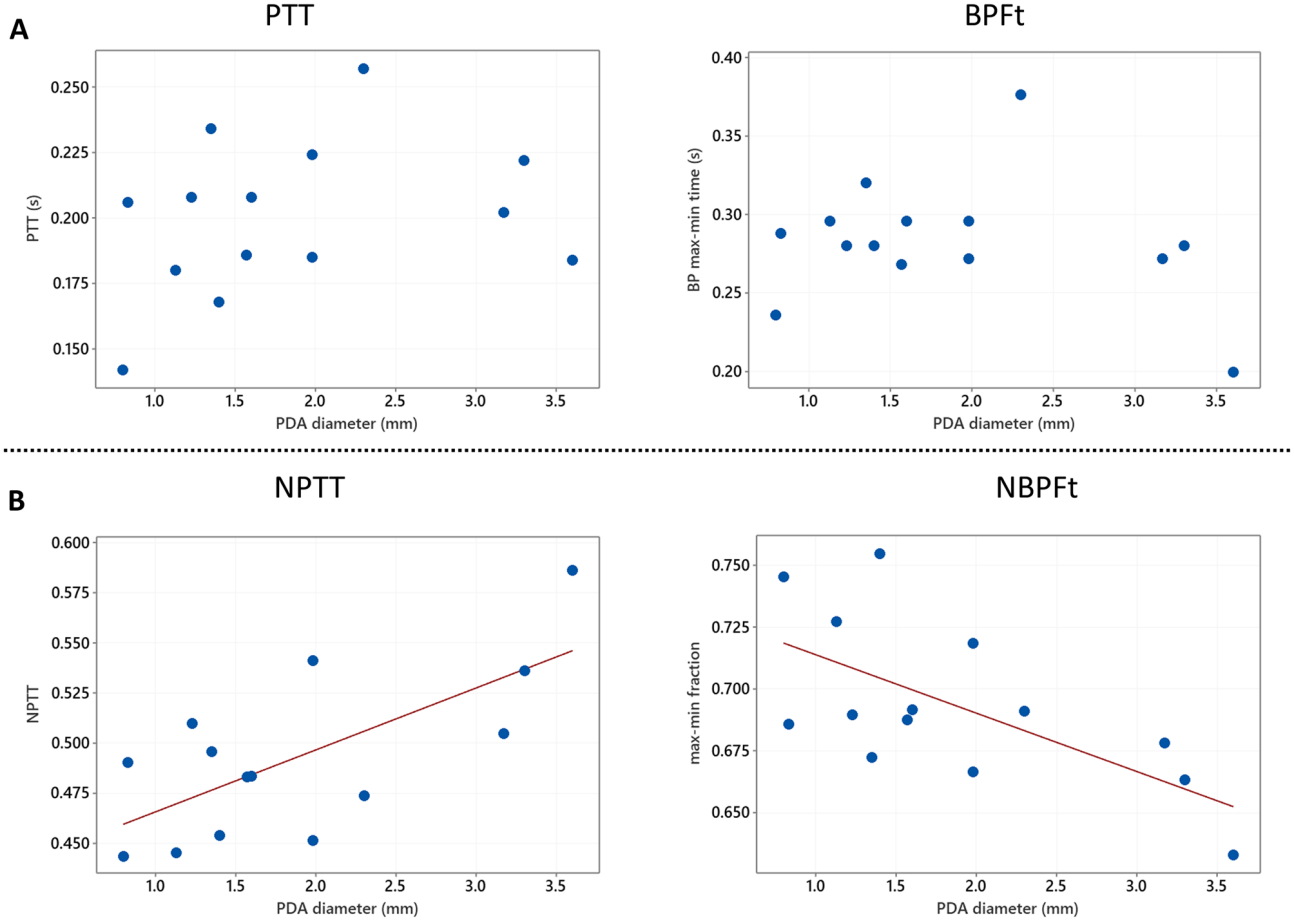
shows the effect of normalising data for HR. Figure 2 **A** shows non-normalised PTT and BPFt plotted against PDA diameter. Figure 2 **B** shows the relationship of HR normalised data (NPTT and NBPFt) plotted against PDA diameter

**Fig. 3:**
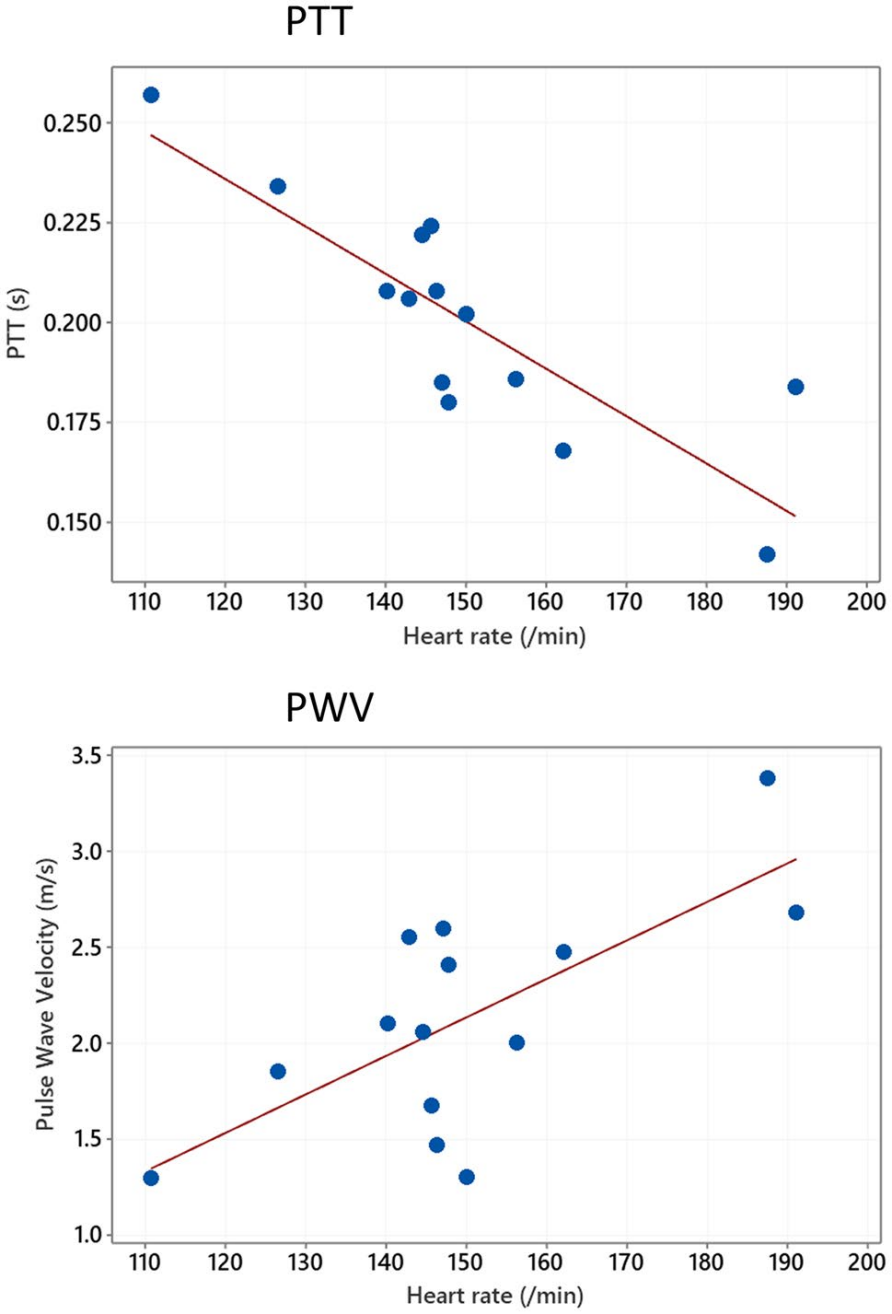
Comparison of PTT and PWV compared with heart rate showing significant relationships

A summary of the BP waveform characteristics and echocardiographic parameters compared with PDA diameter is shown in Table 2 part A. When comparing with PDA diameter, there was no apparent relationship either with PTT or the non-normalised BP waveform measurements (Table 2 part A and Fig. 2A). However, there were statistical significant relationships between the normalised BP waveform measurements as well as NPTT when compared with PDA diameter (Table 2 part A and Fig. 2B). There were statistically significant positive correlations between NPTT and PDA diameter (*r* = 0.69, *P* = 0.007) as well as BP max to min (NBPFt) (*r* = 0.65, *P* = 0.012) and BP min to max (NBPRt) (*r* = 0.71, *P* = 0.005) with PDA diameter; inotropic support did not have any apparent effect in the aforementioned biomarkers. HR was found to have a significant relationship when comparing with PTT, PWV, left ventricular end diastolic diameter/aortic valve diameter ratio (LVEDD:Ao) and E/A wave ratio, but not with PDA diameter (Table 2 and Fig. 3). There was no significant correlation between the distance of the UAC tip from the aortic valve and NPTT (*r* =  −0.04, 95% CI (−0.56, 0.50), *P* = 0.880).

**Table 2:** A Comparison of BP waveform analysis and echocardiographic measurements with PDA diameter showing Pearson’s correlation coefficient confidence interval and P-value (cf: compared with). B. Comparison of BP waveform analysis and echocardiographic measurements with heart rate showing Pearson’s correlation coefficient confidence interval and P-value (cf: compared with)

	***N***	**Correlation coeff ρ**		**95% CI for ρ**	***P*-value**
**A) Variable cf PDA diameter**					
PTT	14	0.28		(−0.294, 0.706)	0.333
***NPTT***	***14***	***0.688***		***(0.247, 0.893)***	***0.007***
BPFt	14	−0.218		(−0.671, 0.354)	0.455
***NBPFt***	***14***	−***0.647***		***(***−***0.877,*** −***0.178)***	***0.012***
BPRt	14	0.337		(−0.235, 0.736)	0.238
***NBPRt***	***14***	***0.705***		***(0.279, 0.899)***	***0.005***
MIVCT	14	0.253		(−0.320, 0.691)	0.382
NMIVCT	14	0.457		(−0.097, 0.795)	0.1
PWV	14	−0.301		(−0.717, 0.273)	0.296
Pulse pressure	14	0.101		(−0.454, 0.600)	0.731
Mean BP	14	−0.09		(−0.593, 0.462)	0.758
Cerebral PI	11	0.512		(−0.127, 0.851)	0.107
LVEDD:Ao	12	0.474		(−0.138, 0.824)	0.12
E/A	13	0.494		(−0.078, 0.822)	0.086
IVRT	13	−0.264		(−0.711, 0.336)	0.384
Tip distance	14	−0.185		(−0.652, 0.383)	0.527
Heart rate	14	0.098		(−0.456, 0.598)	0.738
**B) Variable cf heart rate**					
***PTT***	***14***		−***0.841***	***(***−***0.949,*** −***0.562)***	** < *0.001***
NPTT	14		0.177	(−0.390, 0.647)	0.544
***BPFt***	***14***		−***0.94***	***(***−***0.981,*** −***0.817)***	** < *0.001***
NBPFt	14		0.109	(−0.448, 0.604)	0.711
***BPRt***	***14***		−***0.793***	***(***−***0.932,*** −***0.453)***	***0.001***
NBPRt	14		0.023	(−0.514, 0.547)	0.937
***MIVCT***	***14***		−***0.749***	***(***−***0.916,*** −***0.363)***	***0.002***
NMIVCT	14		0.487	(−0.059, 0.809)	0.078
***PWV***	***14***		***0.701***	***(0.271, 0.898)***	***0.005***
Pulse pressure	14		−0.382	(−0.759, 0.187)	0.178
Mean BP	14		−0.341	(−0.738, 0.231)	0.232
Cerebral PI	11		0.047	(−0.569, 0.629)	0.892
***LVEDD:Ao***	***12***		−***0.713***	***(***−***0.913,*** −***0.236)***	***0.009***
***E/A wave ratio***	***13***		−***0.816***	***(***−***0.943,*** −***0.480)***	***0.001***
IVRT	13		−0.062	(−0.593, 0.506)	0.839
Tip distance	14		0.355	(−0.216, 0.745)	0.212

Data in bold shows significant relations taking account of multiple comparisons using the Benjamini–Hochberg procedure with a false discovery rate of 10%. *PTT* pulse transit time, *NPTT* HR normalised PTT, BP max to min time (BPFt) and normalised (NBPFt), BP min to max time (BPRt) and normalised (NBPRt), isovolumic contraction time (MIVCT) and normalised (NMICVT), *PWV* pulse wave velocity, *PDA* patent ductus arteriosus, *LVEDD:Ao* left ventricular end diastolic diameter/aortic valve diameter ratio, *IVRT* isovolumetric relaxation time, *PI* Pourcelot resistance index, *BP* blood pressure

### Repeatability

 Intra-subject NPTT and BP time difference measurements repeatability was good with coefficient of variation 2.4%, mean difference 0.00 and standard deviation 0.02 for NPTT (Supplement Fig. a) with similar repeatability for BP waveform measurements (Supplement Fig. b and c).

## Discussion

PDA diameter is generally considered the most useful parameter for determining PDA haemodynamic significance [17]. Our study used novel straightforward techniques to measure BP waveform characteristics and interaction between ECG and BP traces in EPIs s and relates them with PDA diameter. Associations were clearest when normalising the characteristics for HR which may be due to the wide range of HR seen (110–191/min). Our approach was found to have good repeatability and potentially allows continuous trend monitoring of the PDA diameter.

We found that increased PDA diameter was associated with longer NBPRt and NPTT and shorter NBPFt. There was no significant association between PDA diameter and IVRT as well as MIVCT which suggests that the isovolumic time intervals are not significantly affected by PDA. The presence of a large PDA makes the heart spend a proportionally longer time in ejection during the cardiac cycle. This is consistent with a previous study and can be explained as PDA increases preload due to increased pulmonary flow and decreases afterload, as it connects the systemic circulation to the low resistance pulmonary circulation [18]. Hence, as the left ventricular volume is increased it takes longer for this to be ejected out of the LV. Moreover, it is known that increased afterload shortens ejection time [19].

Overall the end diastolic time to peak systolic as a proportion of the R-R interval was about 1/3 with longer values being more likely to be associated with larger PDA diameter. In terms of a simple model, a PDA can be considered as a parallel pipe scenario where the ductus and descending aorta are the pipes; the scenario is complicated as the PDA provides a lower resistance channel. Nevertheless considering the simple parallel pipe model, the proportion of the cardiac cycle from peak systole to end diastole would be expected to be reduced with more flow through the PDA as there is an effective ‘steal’ of blood. Alternative modelling approaches have been suggested using the Moens–Korteweg equation; however, it is based on an acoustic approach, and BP is not incorporated [20].

The present study emphasises the importance of reporting HR and developing methods for correcting for HR when echocardiographic [21] and pulse wave characteristics [22] are used to study changes related to PDA in preterm infants. PTT was found to be longer after PDA treatment in a study of ex-preterm infants when looking at group differences [23]; however, the PDA treatment occurred at postnatal age ranging from 10 to 79 days. Moreover, the recordings in our study were from the descending aorta which is central and elastic with lower arterial stiffness compared to peripheral (muscular) arteries. A previous study has demonstrated by using pre- and post-ductal sites’ pulse wave plethysmography that PDA is related to small changes in pulse phase difference between oximetry probes on the left foot and right hand [24]. Compared to this study, our methodology did not require extra probes on the baby nor depend on oximeter plethysmogram processing.

The values for PWV we observed are consistent with a study in children albeit being slightly lower [25]. PDA did not affect the pulse pressure in our study which is consistent with recent reports [26]. Moreover, it is known that the arterial capacitance and not the resistance mainly determines the pulse pressure [27]. The dicrotic limb of the waveform in our population is smoother and does not have the classic appearance of the adult waveform with the prominent dicrotic notch and the bumpy systolic decline and diastolic runoff phase produced by the higher resistance vasculature and the resultant reflective waves. However, BP wave appearance may be related to the BP filter settings on the monitor.

### Limitations

Natural variations of cardiac anatomical landmarks, mode of ventilation, heart size, respiratory cycle phase and angle of the X-ray can affect measurement of distance between the UAC tip and the aortic valve. The monitor filter settings used were standard on our NICU; increasing the BP low pass filter cut-off frequency could achieve a less smoothed waveform and help improve resolution for BP waveform peak and trough as well as better visual feature identification such as the dicrotic notch. We have specified the filter settings used as the monitor signal processing as well as BP measurement site could affect this analysis.

## Conclusions

This pilot study has highlighted the importance of HR when assessing physiological variables related to PDA. We observed significant relationships between PDA diameter and BP waveform characteristics normalised for in extremely preterm infants. The phase difference between ECG and BP waveforms as well as BP waveform characteristics are straightforward to implement using routinely monitored waveforms and so potentially could be incorporated in monitors to allow continuous PDA function assessment.

## Supplementary information

Below is the link to the electronic supplementary material.

Supplementary file1 (DOCX 334 KB)

## Data Availability

Upon reasonable request.

## Abbreviations

BPFt: BP fall time (peak systole to end diastole of BP waveform)
BPRt: BP rise time (end diastole to peak systole of next beat in BP waveform)
BP: Blood pressure
BW: Birth weight
ECG: Electrocardiogram
EPIs: Extremely preterm infants
HR: Heart rate
IVRT: Isovolumic relaxation time
MIVCT: Isovolumic contraction time
NBPFt: BP fall time (peak systole to end diastole of BP waveform) normalised for heart rate
NBPRt: BP rise time (end diastole to peak systole of next beat in BP waveform) normalised for heart rate
NMIVCT: Isovolumic contraction time normalised for heart rate
NPTT: Pulse transit time normalised for heart rate
PDA: Patent ductus arteriosus
PTT: Pulse transit time
PWV: Pulse wave velocity

## Code availability

Upon reasonable request.
