# Supplementary material for: Assessing patent ductus arteriosus in preterm infants from standard neonatal intensive care monitoring
Source: Eur J Pediatr. 2021 Nov 8;181(3):1117–24. doi: 10.1007/s00431-021-04311-9 (PMC8897357; doi:10.1007/s00431-021-04311-9)
Supplement: Supplementary file 1 — Supplementary file1 (DOCX 334 KB) [file 431_2021_4311_MOESM1_ESM.docx]

**Supplementary information**


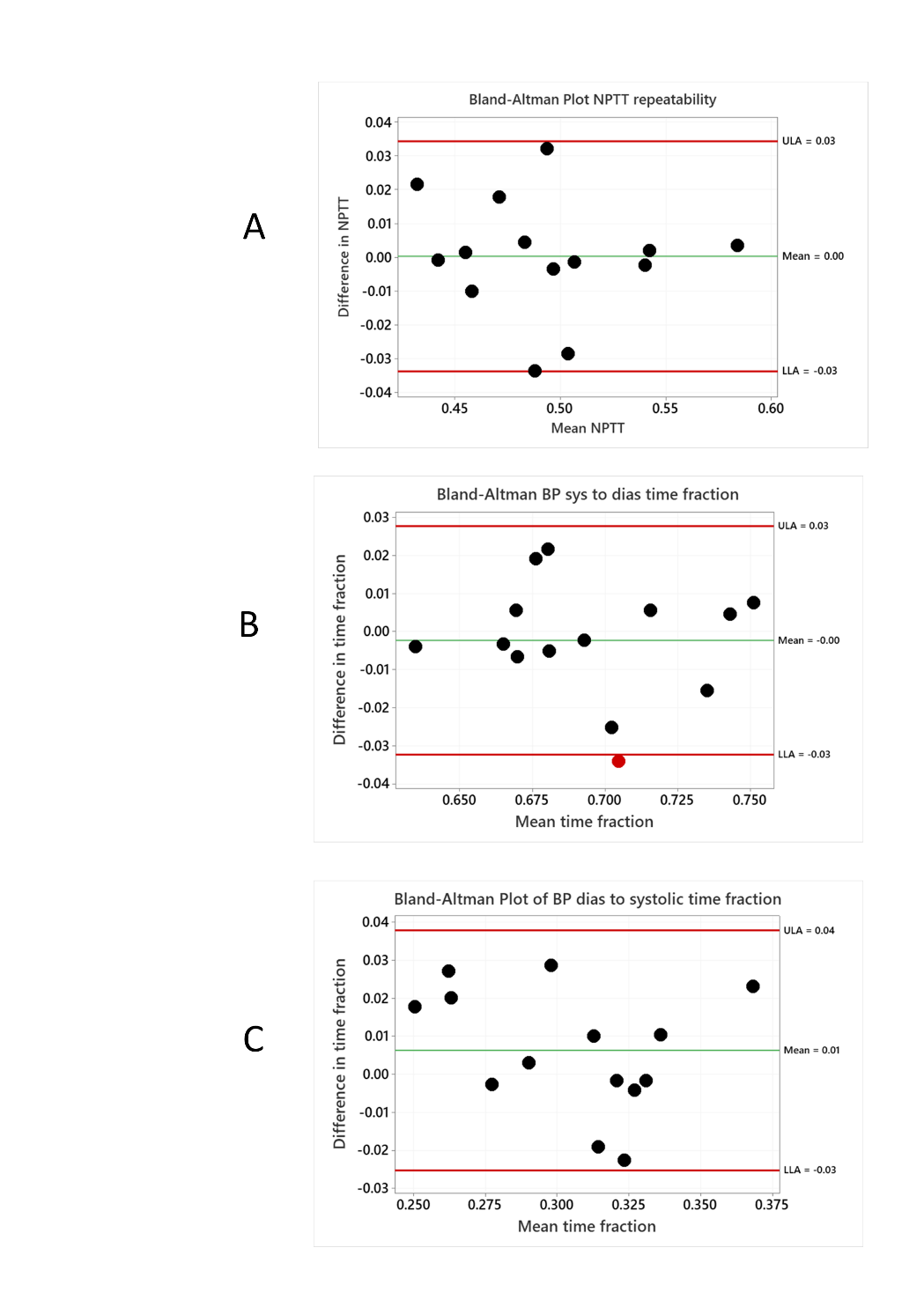


Supplementary graphs, A: plot of the difference between measurements of HR normalised PTT compared with the mean indicating a mean difference of 0.00 with standard deviation of 0.02 and intra-subject coefficient of variation 2.4%. Fig B shows a plot of the HR normalised difference between measurements of BP systole to diastole time (NBPFt) compared with the mean indicating a mean difference of -0.00 with standard deviation of 0.02 and intra-subject coefficient of variation 1.5%. The red point indicates a value outside the limits of agreement. Fig C shows a plot of the difference between measurements of HR normalised BP diastole to systole time (NBPRt) compared with the mean indicating a mean difference of -0.01 with standard deviation of 0.02 and intra-subject coefficient of variation 3.9%. The Bland Altman plots show there was good repeatability between the measurements in the two 10 seconds segments.
